# Stability of Frozen Serum Levels of Insulin-like Growth Factor-I, Insulin-like Growth Factor-II, Insulin-like Growth Factor Binding Protein-3, Transforming Growth Factor *β*, Soluble Fas, and Superoxide Dismutase Activity for the JACC Study

**DOI:** 10.2188/jea.15.S67

**Published:** 2005-05-16

**Authors:** Yoshinori Ito, Kei Nakachi, Kazue Imai, Shuji Hashimoto, Yoshiyuki Watanabe, Yutaka Inaba, Akiko Tamakoshi, Takesumi Yoshimura

**Affiliations:** 1 Department of Public Health, Fujita Health University School of Health Sciences.; 2 Department of Radiobiology/Molecular Epidemiology, Radiation Effects Research Foundation.; 3 Department of Hygiene, Fujita Health University School of Medicine.; 4 Department of Epidemiology for Community Health and Medicine, Kyoto Prefecture University of Medicine Graduate School of Medical Science.; 5 Department of Epidemiology and Environmental Health, Juntendo University School of Medicine.; 6 Department of Preventive Medicine/Biostatistics and Medical Decision Making, Nagoya University Graduate School of Medicine.; 7 Fukuoka Institute of Health and Environmental Sciences.

**Keywords:** Serum Storage, IGFs, sFas, TGF-*β*, SOD

## Abstract

BACKGROUND: Subjects of the Japan Collaborate Cohort Study (JACC Study) gave peripheral blood samples collected between 1988 and 1990. We conducted to investigate whether levels of serum components measured after 9 years of frozen storage are stable or not.

METHODS: To assess the degradation of frozen serum components in the JACC Study, we compared levels of various components (IGF-I, IGF-II, IGFBP-3, TGF-*β*1, sFas, and total SOD activity) between fresh and stored sera collected from other inhabitants. Serum levels of constituents were measured by immunoradiometric assay (IGF-I, IGF-II and IGFBP-3), quantitative enzyme immunoassay (TGF-*β*1), enzyme-linked immuno-adsorbent assay (sFas), and an improved nitrite method (SOD activity).

RESULTS: The coefficients of variation for intra- and inter-assay precisions of the measurements were less than 9%. Levels of IGF-I, IGF-II, IGFBP-3, TGF-*β*1 and sFas in sera after storage for 9 years at -80°C were similar to those of fresh sera newly collected from inhabitants. The distributions of serum IGF-I, IGF-II, IGFBP-3, TGF-*β*1, sFas and SOD activity for specimens collected from different individuals tended to be similar to those of serum levels for frozen specimens collected from different individuals and stored for 9 years.

CONCLUSIONS: There was no statistically significant difference in distribution of measured values of IGF-I, IGF-II, IGFBP-3, TGF-*β*1, and sFas between newly collected sera and frozen specimens stored for 9 years. Thus, measurements of these serum constituents of specimens stored for the JACC Study can be reliably used in nested case-control study.

The Japan Collaborate Cohort Study for Evaluation of Cancer Risk (JACC Study), sponsored by Monbusho (the Ministry of Education, Science, Sports and Culture of Japan), involves more than 127,477 participants living in 45 municipalities all over Japan.^1^^,^^2^ Subjects of the JACC Study completed a survey and gave peripheral blood samples collected from 39,242 registered subjects (aged from 40 to 79 years) between 1988 and 1990. Serum from these samples was separated from blood cells and stored in deep freezers at -80°C until 1999; serum of each participant was divided into 3 to 5 tubes (100 to 500 *μ*L per tube). These serum samples are used to study the relation between serum component levels and the incidence or mortality of cancer or other diseases.

Recently, cancer prevention research has focused on molecular biology related to genes, cytokines and special molecules associated with the promotion or inhibition of the development of carcinogenesis and apoptosis. There were some reports of the relationship investigated between cancer and the following serum constituents: insulin-like growth factor (IGF)-I, IGF-II,^3^^-^^5^ insulinlike growth factor-binding protein 3 (IGFBP-3),^6^ transforming growth factor (TGF)-*β*1,^7^^,^^8^ soluble Fas (sFas),^9^ and superoxide dismutase (SOD) activity.^10^^-^^13^ Although reports indicate that serum levels of constituents such as proteins and minerals are stable in long-term refrigerated storage,^14^^-^^17^ there have been no reports indicating whether serum levels of cytokines, such as IGFs, TGF-*β* and sFas, remain stable after approximately 10 years of storage at -80°C.

In the present study, we examined whether cytokines and other constituents in frozen sera remained stable during long-term storage at -80°C, using stored and fresh serum samples separately collected from other subjects.

## METHODS

### Serum Samples

Approximately 2 liters of pooled sera prepared to evaluate the stability of serum biochemical constituents for the JACC Study was collected from individuals who participated in health check-up programs for workers in certain industries. After centrifugation and filtration 3 times, 1mL samples of the pooled sera were put into 2-ml cups that were sealed with a polypropylene stopper, distributed to laboratories and stored at -80°C beginning in 1988. Fresh sera used for comparison of serum levels between fresh and frozen sera were separately collected from inhabitants of rural Saitama (1999) and Hokkaido (1990) who participated in health check-up programs. Serum samples used for comparison of serum level of inhabitants, aged 40 to 80, were collected from residents of Hokkaido who attended health check-up programs in August 1991 and 1999. Approximately 3mL serum samples were poured into polypropylene cups sealed with a polypropylene stopper, and were stored at -80°C until the time of measurement of components. Reference sera for intra- and inter-assay precisions were used different levels of controlled specimens or pooled sera specially prepared by SRL Laboratory (SRL Laboratory, Hachioji).

### Measurements of serum constituents

We measured serum levels of IGF-I, IGF-II, IGFBP-3, TGF-*β*1, sFas and total SOD activity. All measurements were performed using the same batched-reagent set, by trained staff at a single laboratory (SRL Laboratory, Hachioji). Measurements used for comparison between fresh and stored sera were performed in 1999. Serum component levels of pooled sera were measured using a SMAC auto-analyzer (Technicon Co., Ltd.; for measurements in 1988) and a TBA auto-analyzer (Toshiba K.K.; for measurements in 1994).

Serum levels of IGF-I, IGF-II and IGFBP-3 were measured by immuno-radiometric assay, using commercially available kits (Daiichi Radioisotope Lab., Tokyo)^18^^-^^20^ (Table 1). Serum TGF-*β*1 was measured by quantitative sandwich enzyme immunoassay (ELISA), using commercially available kits (R&D Systems Inc., Minneapolis).^21^^,^^22^ Serum sFas was assayed by enzyme-linked immuno-adsorbent assay (ELISA), using commercially available kits (MBL Co., Ltd., Nagoya).^23^^,^^24^ Serum SOD activity was estimated from the decreasing rate of nitrite produced by hydroxylamine and superoxide anions, based on an improved nitrite method.^25^

**Table 1.** Determination method and its precision when serum levels of IGFs, IGF-BP3, TGF-*β*1, sFas, and SOD activity in serum samples were estimated by the method used in this study.

Item		IGF-I	IGF-II	IGFBP-3	TGF-*β*1	sFas	SOD activity
Assay method		Immuno- radiometric assay (IRMA)	Immuno- radiometric assay (IRMA)	Immuno- radiometric assay (IRMA)	Quantitative sandwich enzyme immunoassay	Enzyme-linked immuno- adsorbent assay (ELISA)	Improved nitrite method (Colorimetric method)

Assay reagents	Company supplied the reagent kit	Daiichi Radioisotope Lab.	Daiichi Radioisotope Lab.	Daiichi Radioisotope Lab.	R&D Systems Inc.	BML Company Ltd.	SRL Lab.

Detection	Ranges (unit)	4-2,000 (ng/mL)	10-1,640 (ng/mL)	0.06-10.10 (*μ*g/mL)	16-2,178 (ng/mL)	1.0 - 10.0 (pg/mL)	0.1 - 10.0 (U/mL)

Precision	Intra-assay(%)	2.15 - 3.53	2.74 - 4.45	3.16 - 4.19	2.67 - 6.79	2.18 - 5.55	4.02 - 6.79
	Inter-assay(%)	1.12 - 4.18	4.23 - 5.53	5.28 - 8.89	4.17 - 6.16	8.24 - 12.30	2.79 - 5.82

Assay precision	Inter-precision for JACC Study	2.30	-	8.74	7.51	7.90	8.77

Reference serum (CV%): Coefficients of variation were caliculated from the mean values of reference sera estimated by each assay for JACC Study samples.

The coefficients of variation (CV) of intra- and inter-assay precisions for each determination were calculated: from 10 determinations of 3 different reference sera for intra-assay precision; and from 5-day determinations of 5 different reference sera for inter-assay precision. Each range of CV values estimated with different reference sera was presented as the lowest and highest mean values. The ranges of inter-assay precision for the JACC Study were represented as the low and high CV values calculated from the reference serum levels estimated in each assay of the JACC Study samples. Paired t-tests were performed to evaluate the mean differences between fresh and frozen sample levels.

Our entire study design, which comprised singular and collective use of epidemiological data and sera, was approved by the Ethical Board at Nagoya University School of Medicine, where the central secretariat of the JACC study is located.

## RESULTS

The range of the assays for reliable measurement of IGF-I, IGF-II and IGFBP-3 in reference sera was 4 to 2,000 ng/mL, 10 to 1,640 ng/mL, and 0.06 to 10.10 *μ*g/mL, respectively (Table 1). The intra- and inter-assay precisions obtained using different reference sera for each determination method was as follows: for the IGF-I assay, 2.15 to 3.53% and 1.12 to 4.18% of the CV values, respectively; for the IGF-II assay, 2.74 to 4.45% and 4.23 to 5.53%; for the IGFBP-3 assay, 3.16 to 4.19% and 5.28 to 8.89%. The range of the assay for serum TGF-*β*1 level was 16 to 2,178 ng/mL; the intra- and inter-assay precisions were 2.67 to 6.79% and 4.17 to 6.16% of the CV values, respectively. The range of the assay for serum sFas level was 5.0 to 50 pg/ml; the intra- and inter-assay precisions were 2.18 to 5.55% and 8.24 to 12.30%, respectively. The range of the assay for serum SOD activity was 0.1 to 10.0 U/ml; the intra- and inter-assay precisions were 4.02 to 6.79% and 2.79 to 5.82%, respectively. Mean day-to-day variations (inter-assay precision) of reference sera estimated at the time of measurements for the JACC Study samples were 2.30% for IGF-I, 8.74% for IGFBP-3, 7.51% for TGF-*β*1, 7.91% for sFas, , and 8.77% for SOD activity.

Table 2 shows the comparison of serum component levels between fresh samples and samples stored for 6 years at -80°C. There were no apparent differences in serum levels of proteins or minerals, although there were decreases in serum levels of organic compounds including bilirubin, lipids and some enzyme activities, but serum levels of uric acid, GOT and LDH activities tended to increase, because of bias due to use of different auto-analyzers.

**Table 2.** Comparison of serum costituent values in pooled serum determined between 1988 and 1994.

Serum component	Year of determination	

	1988	1994
Total protein (g/dL)	7.5	7.1
Albumin (g/dL)	3.8	4.0
Total bilirubin (mg/dL)	0.7	0.5
Urea (mg/dL)	22	21
Uric acid (mg/dL)	4.9	5.7
Creatinine (mg/dL)	1.5	1.3

Total cholesterol (mg/dL)	209	189
Triglyceride (mg/dL)	121	109

Sodium (mEq/L)	146	146
Potassium (mEq/L)	4.4	4.4
Chloride (mEq/L)	108	102
Inorganic phosphate (mg/dL)	3.9	3.9
Calcium (mg/dL)	8.5	8.0

GOT (IU/L)	16	19
GPT (IU/L)	14	14
LDH (IU/L)	64	75
ALP (IU/L)	52	39
CHE (IU/L)	4,074	3,520
*γ*-GTP (IU/L)	46	42
LAP (IU/L)	94	77
Amylase (IU/L)	28	23

Autoanalyzer	SMAC	TBA
	(Technicon Co., Ltd.)	(Toshiba K.K.)

1988: data estimated from fresh pooled serum at the time of preparation.

1994: data estimated from pooled serum stored during a 6-year storage at -80°C.

Serum values of IGF-I, IGF-II, IGFBP-3, TGF-*β*, and sFas in individual sera after storage for 9 years at -80°C, which were separately collected from Hokkaido inhabitants in 1990, were similar to those of fresh sera newly collected from Saitama healthy inhabitants in 1999 (Table 3). In a study of other serum samples collected from 100 healthy individuals (46 males and 64 females) in 1991 and 1999, and stored at -80°C until 2000, there tended to be similarity in distribution of serum values of IGF-I, IGF-II, IGFBP-3 and sFas between serum samples collected in 1991 and 1999 from different individuals, although those of TGF*β*1 and SOD activity tended to change during storage (Table 4).

**Table 3.** Comparison of serum levels of certain cytokines and SOD activity between fresh and frozen samples.

Sample	IGF-I (ng/mL)	IGF-II (ng/mL)	IGFBP-3 (*μ*g/mL) TGF-*β*1 (ng/mL)		sFas (ng/mL)
Fresh sample	199.8 (21.5)	665.7 (50.9)	3.01 (0.11)	32.99 (3.36)	1.92 (0.23)
N	10	10	10	10	10

Frozen sample	186.2 (12.7)	616.3 (29.7)	3.12 (0.25)	30.44 (2.43)	1.77 (0.22)
N	21	21	21	20	19

Probability	p =0.64	p =0.48	p =0.64	p =0.55	p =0.81

Fresh sample: determination at the time of serum collection in 1999 .

Frozen sample: determination of serum samples collected in 1990 after 9-year storage at -80°C.

**Table 4.** Comparison of serum levels of IGFs, IGFBP-3, TGF-*β*1, sFas, and SOD activity in 100 inhabitants (46 males and 64 females, aged 39-78) collected between 1991 and 1999.

Component	Collected year			Mean differences
		1991	1999	
IGF-I (ng/mL)	Mean	167	162	-5 (-3.0%)

	25%	130	120	
	50%	160	150	
	75%	200	200	

IGF-II (ng/mL)	Mean	649	652	3 (-0.5%)

	25%	570	560	
	50%	630	660	
	75%	728	750	

IGFBP-3 (*μ*g/mL)	Mean	3.09	3.03	-0.06 (-1.9%)

	25%	2.72	2.55	
	50%	3.09	3.07	
	75%	3.52	3.51	

IGF-*β*1 (ng/mL)	Mean	32.3	36.9	4.6 (14.2%)

	25%	22.5	31.6	
	50%	32.0	36.7	
	75%	43.6	42.2	

sFas (ng/mL)	Mean	2.44	2.64	0.20 (8.2%)

	25%	1.40	1.6	
	50%	1.70	1.85	
	75%	2.00	2.2	

SOD Activity (U/mL)	Mean	2.9	2.5	-0.4 (-13.8%)

	25%	1.63	1.8	
	50%	1.9	2.1	
	75%	2.2	2.5	

Data represented as mean (mean value) and ranges (25%, 50% and 75%).

Mean difference: difference value = 1999-level - 1991-level.

Difference precentages (%) = difference value/ 1991-level.

## DISCUSSION

In the JACC Study, sera collected from 39,242 subjects and separated from blood cells were stocked in deep freezers at -80°C until 1999; serum of each participant was divided into 3 to 5 tubes (100 to 500 *μ*L per tube). We were unable to assess the stability of serum samples stored for about 10 years, because the volume of serum samples for the JACC Study was insufficient for measurements of many constituents using different various protocols. Therefore, we evaluated the stability of frozen and stored sera using serum samples separately collected from other inhabitants and pooled serum.

Results of this study, in which values were compared between fresh and frozen samples of pooled serum prepared for quality control of determination of JACC Study samples, demonstrate that serum levels of proteins such as albumin and total protein tend to remain steady during frozen storage for several years.

Some reports indicate that there is little change in serum levels of constituents such as proteins, minerals, glucose and uric acid during storage at -70°C,^14^ although serum levels of creatinine and lipids such as triglyceride tended to decrease during storage for 6 years at -80°C in this study. The difference in enzyme activities in the present study may be due to the estimation methods used for each auto-analyzer, although it has been reported that serum AST (GOT) activity changes during storage.^14^^,^^15^ It has also been reported that the plasma protein fraction can be safely used after storage for 5 years at room temperature.^17^ There have been no previous detailed reports about the stability of cytokines such as IGFs, TGF-*β*1 and sFas in frozen serum samples examined after long-term storage. In the present study, the mean values of cytokines such as TGF-*β*1 in sera separately collected from inhabitants varied over a range of about 14% after 9 years of storage at -80°C. However, we also obtained that serum values of other cytokines such as IL-6 (but not TNF-*α*) tended to decrease (more than 60%) after 9 years of storage at -80°C, in comparison between fresh and frozen samples. The range of variation was similar to the reported coefficients of variation for determinations of IGFs, TGF-*β*, sFas and SOD activity: 1.1 to 7.3% for intra-assay, and 1.6 to 11.7% for inter-assay.^18^^-^^25^ Moreover, in the present study, serum SOD activity was stable during long-term storage at -80°C. In previous studies of SOD activity, there was no significant change related to storage time or temperature,^12^^,^^26^ erythrocyte-SOD activity was unstable,^27^ and protein levels were unusually stable.^28^

The present results indicate that serum levels of IGF-I, IGF-II, IGFBP-3, sFas, and TGF-*β*1 remain stable during long-term storage at -80°C, because distributions of serum levels of these constituents were nearly equal between fresh and frozen specimens separately collected from different inhabitants. They also suggest that SOD activity is a useful biomarker for cancer prevention research such as a nested case-control study.

## MEMBER LIST OF THE JACC STUDY GROUP

The present investigators involved, with the co-authorship of this paper, in the JACC Study and their affiliations are as follows: Dr. Akiko Tamakoshi (present chairman of the study group), Nagoya University Graduate School of Medicine; Dr. Mitsuru Mori, Sapporo Medical University School of Medicine; Dr. Yutaka Motohashi, Akita University School of Medicine; Dr. Ichiro Tsuji, Tohoku University Graduate School of Medicine; Dr. Yosikazu Nakamura, Jichi Medical School; Dr. Hiroyasu Iso, Institute of Community Medicine, University of Tsukuba; Dr. Haruo Mikami, Chiba Cancer Center; Dr. Yutaka Inaba, Juntendo University School of Medicine; Dr. Yoshiharu Hoshiyama, Showa University School of Medicine; Dr. Hiroshi Suzuki, Niigata University School of Medicine; Dr. Hiroyuki Shimizu, Gifu University School of Medicine; Dr. Hideaki Toyoshima, Nagoya University Graduate School of Medicine; Dr. Shinkan Tokudome, Nagoya City University Graduate School of Medical Science; Dr. Yoshinori Ito, Fujita Health University School of Health Sciences; Dr. Shuji Hashimoto, Fujita Health University School of Medicine; Dr. Shogo Kikuchi, Aichi Medical University School of Medicine; Dr. Akio Koizumi, Graduate School of Medicine and Faculty of Medicine, Kyoto University; Dr. Takashi Kawamura, Kyoto University Center for Student Health; Dr. Yoshiyuki Watanabe, Kyoto Prefectural University of Medicine Graduate School of Medical Science; Dr. Tsuneharu Miki, Kyoto Prefectural University of Medicine Graduate School of Medical Science; Dr. Chigusa Date, Faculty of Human Environmental Sciences, Mukogawa Women’s University ; Dr. Kiyomi Sakata, Wakayama Medical University; Dr. Takayuki Nose, Tottori University Faculty of Medicine; Dr. Norihiko Hayakawa, Research Institute for Radiation Biology and Medicine, Hiroshima University; Dr. Takesumi Yoshimura, Institute of Industrial Ecological Sciences, University of Occupational and Environmental Health, Japan; Dr. Akira Shibata, Kurume University School of Medicine; Dr. Naoyuki Okamoto, Kanagawa Cancer Center; Dr. Hideo Shio, Moriyama Municipal Hospital; Dr. Yoshiyuki Ohno, Asahi Rosai Hospital; Dr. Tomoyuki Kitagawa, Cancer Institute of the Japanese Foundation for Cancer Research; Dr. Toshio Kuroki, Gifu University; and Dr. Kazuo Tajima, Aichi Cancer Center Research Institute.
